# Effects of Diet Consistency on Rat Maxillary and Mandibular Growth within Three Generations—A Longitudinal CBCT Study

**DOI:** 10.3390/biology12091260

**Published:** 2023-09-20

**Authors:** Ioannis A. Tsolakis, Christos Verikokos, Despoina Perrea, Paula Perlea, Konstantina-Eleni Alexiou, Zafeiroula Yfanti, Ioannis Lyros, Maria Georgaki, Erofili Papadopoulou, Apostolos I. Tsolakis

**Affiliations:** 1 Department of Orthodontics, School of Dentistry, Faculty of Health Sciences, Aristotle University of Thessaloniki, 54124 Thessaloniki, Greece; 2 Department of Orthodontics, Case Western Reserve University School of Dental Medicine, Cleveland, OH 44106-7342, USA; 3 Second Department of Surgery, “Laikon Hospital”, School of Medicine, National and Kapodistrian University of Athens, 15772 Athens, Greece; 4 Laboratory of Experimental Surgery and Surgical Research, School of Medicine, National and Kapodistrian University of Athens, 15772 Athens, Greece; 5 Department of Endodontics, Carol Davila University of Medicine and Pharmacy, 050474 Bucharest, Romania; 6 Department of Oral Diagnosis & Radiology, School of Dentistry, National and Kapodistrian University of Athens, 10679 Athens, Greece; 7 Department of Orthodontics, School of Dentistry, National and Kapodistrian University of Athens, 15772 Athens, Greece; 8 Department of Oral Medicine & Pathology and Hospital Dentistry, School of Dentistry, National and Kapodistrian University of Athens, 11527 Athens, Greece

**Keywords:** growth, soft diet, hard diet, maxilla, mandible, condylar process

## Abstract

**Simple Summary:**

It has been established that a variety of factors, including the environment, influence craniofacial growth. Environmental alterations are regarded to have the potential to impact maxillary and mandibular growth. Numerous studies have partially addressed the question of how diet consistency influences maxillary and mandibular growth in one generation of rats by suggesting that diet consistency may lead to variable masticatory forces that affect mandibular growth. There has not been any research carried out yet that looks at potential qualitative and quantitative growth changes in the maxilla and mandible throughout the course of different generations using 3D imaging technology and wistar rats as the experimental animal of choice. The current study assessed the effects of various meal compositions on the growth of the mandible across three generations. The findings of this study suggest that a soft diet may be to blame for decreased maxillary and mandibular growth, and this genetic information may be passed down through the generations.

**Abstract:**

Background: In this study, wistar rats were used to examine the impact of diet consistency on maxillary and mandibular growth over three generations. Methods: In this investigation, a breeding sample of 60 female and 8 male wistar rats was used. Measuring was only performed on female animals. The first generation’s primary breeding sample consisted of 20 female wistar rats that were 30 days old and 4 male rats that were also 30 days old; two subsequent generations were created from these animals. At the age of 100 days, CBCTs were collected of all male rats. Twenty-eight craniofacial landmarks were selected for the linear measurements on stl format extracted from the DICOM files. A Bonferroni test was performed for the statistical analysis. Results: Means of measurements of all soft diet groups compared to corresponding measurements of the hard diet groups were significantly different. According to linear measurements, there was statistical difference on the maxillary measurements between the soft diet groups of the first and third generation, while the rest did not appear to have any statistical difference. There was significant difference for the mandibular dimensions only when the first generation soft diet group was compared with the third generation soft diet group. Conclusions: Food consistency has a significant impact on the growth and development of the maxilla and mandible. Soft diet habits may result in retrognathic mandible, and narrower maxilla.

## 1. Introduction

The craniofacial system grows as a result of environmental and inherited variables [1,2]. It is widely acknowledged that muscle loading forces contribute to bone formation and growth [3]. The orofacial region is one of the most important muscular systems since it is required for feeding in vertebrates. Jaw movement and stresses on the orofacial region are intimately related to mandibular growth [4]. A number of developmental alterations in the craniofacial area, especially the mandible, appear to be caused by mastication as one of the environmental influences [5,6]. This could be the explanation for the average rise in malocclusions observed in industrial cultures throughout the 20th century [7,8]. According to recent research, the maxilla’s morphology, which deforms less in those who can generate proportionately bigger temporalis muscle forces, controls how much force is transmitted from the mastication process to the rest of the skull [9]. Additionally, recent studies have revealed a connection between mandibular muscle force and shape. Consequently the total shape of the mandible may be due to some extend masticatory muscle force and, mandibular loading [10]. It is suggested that the decline in functional constraints has led to the increased form variation seen in modern/urban populations [11].

Craniofacial growth is primarily influenced by genetic factors, but it also can be affected to a significant degree by environmental factors such as dietary conditions, status of physical activity and health condition. The major contemporary genetic theories, trying to explain the determinants of craniofacial growth, imply that either the bone itself, the cartilage or the soft tissue matrix surrounding the hard tissues mainly control craniofacial growth, but it is generally accepted that environmental factors contribute to the final expression of gene information. The main genetic and, to a lesser extent, functional and environmental, variables affect the growth of the face. It is believed that force and function have a close relationship. The growth process is influenced by a variety of factors, including genetic control, differential cellular responses, neurotrophic control, membrane control, oxygen tension, bioelectric potentials, pH levels, temperature effects, chalone-like inhibitors and stimulators, cyclic AMP, vasomotor control, nutrition, and enzymatic and hormonal factors. It is crucial to understand the difference between primary and secondary, or supporting, elements. Facial growth is thought to be influenced by specific combinations of all the aforementioned elements, which regulate the size, shape, and position of the faces in the craniofacial skeleton [12]. Compared to populations from non-industrialized societies, the human population of a contemporary civilization experiences more severe malocclusions. Modern food practices put less strain on the masticatory muscles than earlier dietary practices, which has been linked to an increase in malocclusion frequency. Researchers are therefore interested in how food consistency affects craniofacial growth. Rats were used as experimental animals in the majority of experimental studies to examine the relationship between diet consistency and growth. Rats are frequently chosen as experimental animals because they are tiny, readily kept, express little social anxiety, have a brief lifespan, and have well-known genetic backgrounds and growth patterns that are similar to those of humans [13,14,15,16,17,18,19,20,21,22,23]. Genetics has a significant and well-established effect on both the occlusion and craniofacial growth. Similar craniofacial growth trends have been observed in many family generations. This led to the conclusion that some heritability exists for craniofacial growth. Additionally, it is acknowledged that the environment can influence and modify genetic information [24,25].

Intracortical bone remodeling (or simply remodeling) is the process of existing cortical bone being resorbed and replaced as a result of the coordinated actions of osteoclasts and osteoblasts. As a result of this procedure, secondary osteons, which are cylinder-shaped structures, are produced. Cross-sectional images of secondary osteons show concentric lamellae and a cement line surrounding them. The resorption phase of the remodeling process releases mineral reserves to maintain mineral homeostasis, but it also causes mechanical deformation that results in the formation of microcracks. Microcracks have been associated with both large mechanical deformations (high strain) and repetitive cycles of loading. As a result, regions of the skeleton under more demanding loading conditions ought to have higher rates of remodeling since those regions ought to experience more microdamage. It is less clear if greater remodeling may be caused by high strain or cyclical loading when the load situation is unclear. The current body of literature contains controversial findings that support the theory that increased remodeling may be more likely to occur under conditions of cyclical loading rather than high strain [26,27,28,29,30,31,32].

With the aid of molecular biology, different epigenetic mechanisms that extend from the cell membrane to the nucleus and are made up of intracellular macromolecular chain reactions are now understood. The extracellular setting and the nucleus exchange information in this manner. As a result of its ability to recognize and react to mechanical stimuli, the osteocyte network is crucial in initiating bone remodeling. In addition, the form of the cells can be altered by loading the tissues. As a result, processes are engaged that even alter the methods by which the genome acts, leading to deformation of the intracellular material, including the cytoskeleton. Through this mechanism the epigenetic information can be inherited [33,34,35].

As documented by Odman et al. in 2008, a 7-month interval with reduced masticatory requirements in a soft diet group throughout adolescence and early adulthood led to smaller mandibles. Through morphometric research, it was discovered that the angular process area and the condylar process inclination differ significantly. Sprague Dawley rats were utilized as the experimental animals in this study [36]. Trabecular bone had a higher degree of mineralization than cortical bone, according to a 2007 Tanaka et al. study on wistar rats. The anterior mandibular region has higher levels of mineralization than the posterior mandibular region. The soft diet group showed a higher level of mineralization than the hard diet group in those two locations. The trabecular bone of the condyle in the hard diet group had more mineralization than the soft diet group [37]. Similar hypotheses were made by Grunheid et al. in 2011, but they used New Zealand White rabbits as their experimental animals. Their findings showed that the remodeling rate is not significantly affected by a moderate reduction in masticatory functional load [38]. By utilizing wistar rats as their experimental animals in 2022, Tsolakis et al. pointed out that a soft diet led to a smaller condyle and a decreased angle of the jaw as well as the body of the mandible [39].

The existing literature demonstrates that, compared to chewing soft food, chewing hard food improves practically all physiological masticatory parameters, muscular coordination, and masticatory side changes. The association between mastication and general health problems, such as obesity and diminished cognitive function, as well as a more variable and symmetrical weight on the craniofacial structures that affects their growth and wellness, may be explained in part by these findings [40].

Robles RA developed Cone Beam Computer Tomography (CBCT) in 1982 for use in angiography [41]. In a manner similar to Computed Tomography (CT), CBCT rotates around the object of interest while taking several images, creating a three-dimensional (3D) volume. However, CBCT uses a volumetric approach that only requires one rotation, resulting in lower radiation exposure. Recent advancements in CBCT imaging techniques have made it possible to expose patients to less radiation than with conventional two-dimensional radiography. This machine gave the ability to represent the skull with less radiation but with high accuracy [42].

Numerous studies with varying outcomes from that expectation have addressed the subject of how diet consistency influences maxillary and mandibular growth within one generation. There is not much research that has examined potential maxillary and mandibular alterations throughout generations, and only one equivalent study in rats was conducted in the past.

The aim of this study is to examine the impact of different food consistencies on maxillary and mandibular growth within three generations through 3D imaging technology using Cone Beam Computed Tomography (CBCT).

## 2. Materials and Methods

Prior to beginning this investigation, the General Directorate of Veterinary Policy of the prefecture of Attica in the Hellenic Republic received approval from the Institutional Review Board. 1405 is the approval code. Subjects were chosen from the National and Kapodistrian University of Athens School of Medicine’s Laboratory of Experimental Surgery and Surgical Research in Athens, Greece. Eight male and sixty female Wistar rats were employed in the study’s total sample. Male rats were solely utilized in this study for reproduction; all measurements were performed on female rats. The identification of the specimen was not able during all measurements for blinding reasons.

**Figure 1:**
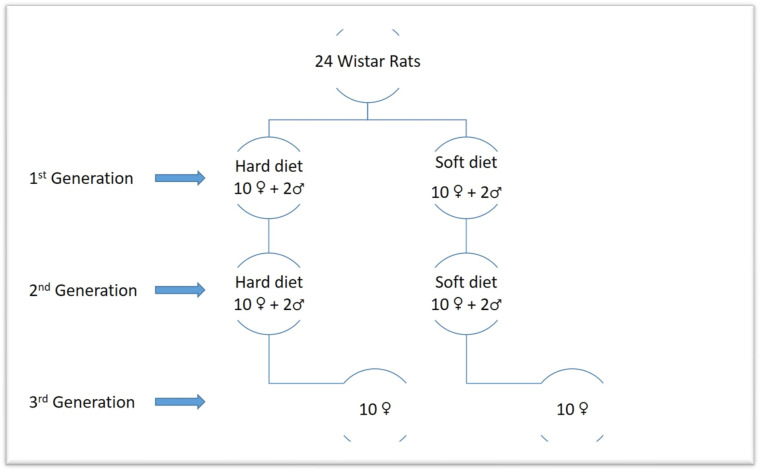
Graphic scheme of the generations.

**Figure 2:**
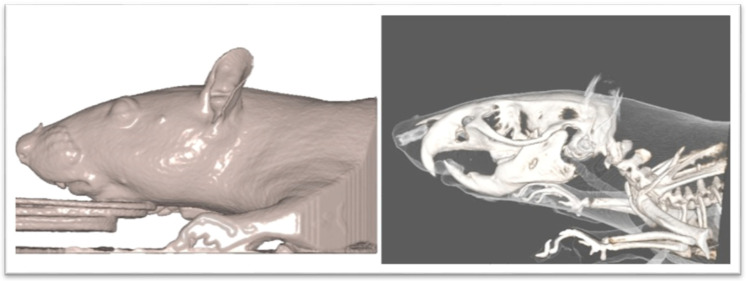
CBCT image of rat.

A power analysis was carried out to determine the results’ power significance based on a prior study [43]. According to the power analysis, a minimum sample size of 10 animals per group will result in a 95% confidence level. Thus, it was decided to choose 20 animals for each generation of this study, 10 for the soft diet group and 10 for the hard diet group. The main sample consisted of 20 female Wistar rats that were 30 days old and 4 male rats. The first generation is made up of the 20 females. By using computer-generated randomness, these were divided into two equal groups of 10 females each. The female rats were fed a soft diet for 30 days in the first group (S1) and a hard diet for 30 days in the second group (H1). Additionally, two males of the same age were fed hard food, while the other two received soft food. Rats from each relevant group were combined on experimental day 31 to facilitate reproduction. After the ablactation phase, the female rats of the first generation were separated from their offspring, and on the 70th day of the experiment, CBCTs were taken before the sacrifice. As a result, we produced two more groups of the second generation, each of which had ten female rats picked at random from the first generation’s progeny. The new groups were distinguished from the old ones using new indicators denoting those who had a soft diet (S2) and those who received a hard diet (H2). Additionally, for the goal of research reproduction, two male rats from each descendant group of the first generation’s soft and hard diets were kept in the experiment. The second generation male and female rats were merged to enhance reproduction at 41 days following each relevant group. After the ablactation phase, the female rats of the second generation were separated from their offspring, and on the 150th day of the experiment, CBCTs were taken before the sacrifice. Two groups of female rats, representing the third generation, were created from the offspring of the second generation, each group consisting of 10 randomly chosen animals. The hard diet group (H3) and the soft diet group (S3) made up the third generation. Additionally, two male rats from each descendant group of the second generation’s soft and hard diets were kept in the experiment for the purpose of breeding. Next, 41 days after the third generation’s relevant groups’ female and male rats were mixed to facilitate reproduction. After the ablactation phase and before euthanasia (on the 230th trial day), the female rats of the third generation were isolated and randomly chosen from their offsprings. CBCTs were also taken (Figure 1 and Figure 2).

The standard rat diet (R34; Lactamin) was administered to the hard diet groups as hard pellets. For the soft diet groups, the standard diet was crushed and mixed in specified ratios with water (2 parts food:5 parts water). To eliminate bulky objects that would induce excessive chewing, the bedding material in the cages of this group was sorted.

**Figure 3:**
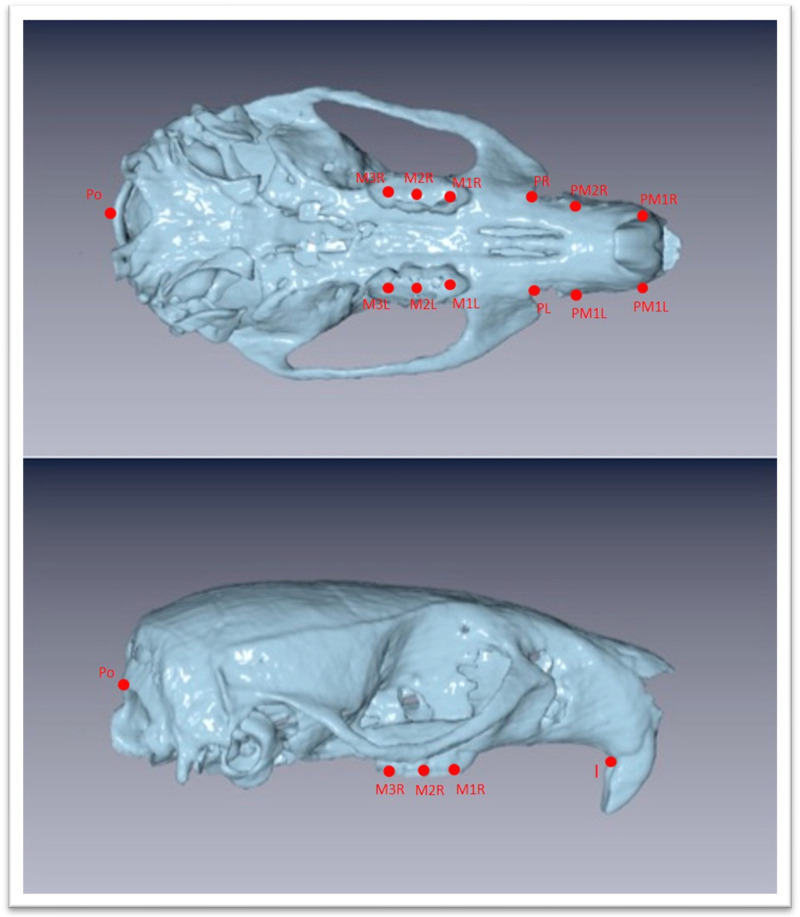
Three-dimensional images of the maxilla extracted as stl file from the DICOM files with chosen landmarks. Landmarks are defined in Table 1.

**Figure 4:**
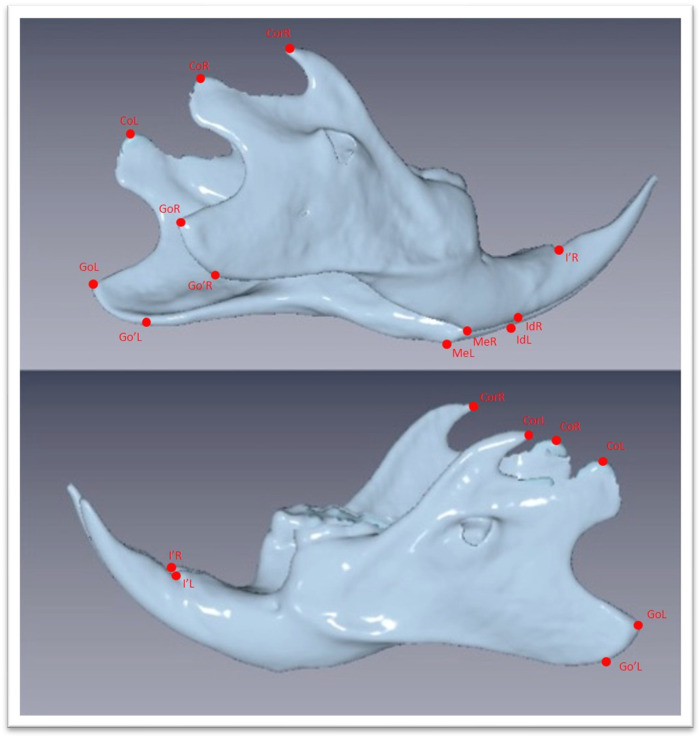
Three-dimensional images of the mandible extracted as stl file from the DICOM files with chosen landmarks. Landmarks are defined in Table 1.

The Νewtom VGi evo^TM^ Cone Beam Computed Tomography (Newtom, Bologna, Emilia-Romagna, Italy) was used. All the animals were sedated in order to be stable during the procedure. Since the head of the wistar rats are small objects, we wanted the size of FOV to be as close as it could be in the skull size, we used the option of the Newtom VGi evo^TM^ to scan smaller objects like a dental cast (Figure 1). Once the DICOM files were created they were uploaded to the Viewbox software (Version 4.1.0.12). From those DICOM files we were able to extract stl files of each skull (Figure 3 and Figure 4). Color mapping was used in order to extract maxilla and mandible from the skull. Twenty-eight craniofacial landmarks were selected for the linear measurements. (Table 1) Eleven measurements were selected for the transverse analysis of the maxilla and one measurement for the analysis on the vertical plane. For the mandible, fourteen measurements showed the differences on the horizontal plane, four measurements showed the differences on the vertical plane, and four measurements indicated the differences on the transverse plane. Once the stl files of each jaw were extracted, they were digitized. (Table 2, Figure 3) All data were imported into a Microsoft Excel spreadsheet (Microsoft Corporation, Redmond, WA, USA) and statistical analysis was performed using SPSS Version 22 (IBM Corporation, Armonk, NY, USA).

### Statistical Analysis

Utilizing intraclass correlation on 20 randomly chosen participants, whose data were re-measured three weeks later, the operator’s reliability was determined.

Regression analysis were utilized for the linear measurements to assess differences linked to diet and generation. On each variable, the effects of diet, generation, and their interactions were regressed. When the normality requirement for the residuals was violated, quantile regression was used. Multiple comparisons were adjusted for using the Bonferroni technique. A statistical significance level of =5% was used for the analysis.

## 3. Results

The effect of food consistency on maxillary and mandibular growth throughout one, two, and three generations was examined in this study. Twenty-four rats were divided into two groups for the current study: the hard diet group and the soft diet group. All rat species reproduced after 30 days of growth. This process took place in order to create three generations. Using CBCTs, we examined how dietary physical consistency affected the growth of the maxilla and mandible. Sixty female rats made up the sample. An excellent level of agreement between all measurements was produced by the intra-observer variability.

### 3.1. Maxillary Measurements

The linear measurements that indicated the transverse analysis of the maxilla were PR–PL, PM1R–PM1L, PM2R–PM2L, M1R–M1L, and M2R–M2L, and M3R–M3L. The horizontal differences were expressed from the I-Po measurement (Table 3 and Table 4). As shown in Table 5, there were no significant differences between all soft diet groups when they were compared with the hard diet groups in PR–PL, PM1R–PM1L, PM2R–PM2L linear measurements. There were no significant changes on M1R–M1L of the first generation soft diet group when they were compared to the hard diet groups, while there were significant changes on M2R–M2L, M3R–M3L. There were significant differences in M1R–M1L, M2R–M2L, M3R–M3L measurements of second and third generation soft diet groups in comparison with hard diet groups. There were no significant changes between all hard diet generations due to measurements. Between the first and second generations of the soft diet groups, as well as between the second and third generations of the soft diet groups, the linear measurements did not reveal any significant differences. Between the first generation and the third generation of the soft diet group, there were significant alterations in M1R–M1L, M2R–M2L, M3R–M3L measurements (Table 5).

### 3.2. Mandibular Measurements

The mandibular measurements that indicated horizontal differences were Go’R–MeR, GoR–MeR, CorR–MeR, CoR\go’R–MeR. CoR–MeR, CoR–IdR, CoR–I’R, Go’L–MeL, GoL–MeL, CorL–MeL, CoL\Go’L–MeL, CoL–MeL, CoL–IdL, CoL–I’L. The measurements of vertical analysis were CoR–GoR, CoR–Go’R, CoL–GoL, CoL–Go’L and for the transverse analysis were CoL–CoR, Go’L–Go’R, MeL–MeR, IdL–IdR. (Table 3 and Table 4) There were significant differences between all soft diet groups when they were compared with the hard diet groups. There were no significant changes between all hard diet generations due to linear measurements. The measurements showed significant differences only on CoR–MeR, CoR–IdR, CoR–I’R, CoL–MeL, CoL–IdL, CoL–I’L and CoL\Go’L–MeL between all possible comparisons of the soft diet groups. The rest of the linear measurements for soft diet groups showed no significant differences between the first and second generation; conversely, the statistical analysis showed differences between the first and the third generation of the soft diet group as significant as between the second and third generations (Table 5).

## 4. Discussion

Previous studies have looked over the influence of diet consistency on growth but only two of them exceeded the one-generation as period of time. All of the studies used lateral cephalometric X-rays or 3D imaging (micro-CT). Three-dimensional imaging cone beam computed tomography gave us the opportunity to have a 3D image of the whole skull of live animals with no risk of losing any hard tissue due to the process of sacrificing the rats and extracting the skull from the body. Furthermore, it is impossible to examine the facial dimensions of a live rat in a micro-CT.

Yamamoto found that food consistency affects the bone appositional pattern at growth site in palatal region of the maxilla [44]. In 1997 Ulgen et al. found that maxillary width have been reduced by examining dry skulls [45]. In 2002 Katsaros et al. examined dry skulls and found that the dental arch was affected. Soft diet groups had narrower arch in the third molar region. Premaxilla and frontal bones were narrower as well [46]. Our study showed that premaxilla measurements had no significant changes. The anterior part of maxilla showed no significant differences between all possible comparisons. The dental arch was becoming narrower in all three generations of soft diet group.

Kiliaridis et al. found that the growth rate in the gonial angle of mandible was increased in the hard diet groups but the angle between occlusal and mandibular plane was decreased in the hard diet groups [47]. In 2002, Maki et al. employed linear measurements on X-rays and discovered that the hard diet groups had higher ramus heights as well as condylar process and coronoid process heights. They found that there was no discernible change in mandibular length between the hard diet and soft diet groups. [48]. Five years later, Abed et al. reported that the anterior corpus length, the ramus height, and the bigonial width were increased in the hard diet groups [49]. Hichijo et al. concurred with the earlier investigations that the hard diet groups’ ramus height increased. While on the contrary the gonial angle and the ramus angle were decreased in the hard diet groups. Mandibular length, mandibular base length, and coronoid process height did not significantly differ between the hard diet and soft diet groups [50]. Recent research by Tsolakis et al. revealed that all measurements corresponding to the mandibular posterior height and length showed significant differences. In the soft diet group, there was a reduction in length and posterior height and any differences in the condyle, the mandibular angle, and the mandibular body were revealed by the morphometric superimposition [39]. The present three dimensional study showed that mandibular morphology changed on horizontal and vertical planes when soft diet groups were compared to hard diet groups, respectively but there was no difference on the transverse plane. From the comparisons between the soft diet groups, it was found that there was significant differences mainly on the horizontal linear measurements that included condylar points between the first generation and the second generation. From the comparison of second and third generation, there were significant differences for all horizontal measurements. At last, comparison of the first and third generations of the soft diet groups showed differences on horizontal as long as on vertical plane.

There are only two studies in the literature that looked over the maxillary and mandibular changes within different generations. Only one of these studies used 3D imaging technology (microCT) to represent the anatomical structures but in this study, mice were used as experimental animals. To be more specific, Hassan et al. examined the variations in craniofacial morphology over 15 generations of mice fed either a soft diet or a hard diet in 2020 [51]. The animals were sacrificed before their skull dimensions were examined with the micro-CT technique. This is in contrast to our method that examined skull dimensions in live rats, avoiding any bony and muscular distraction as a result of skull separation. They found that short-term soft diet consumption resulted in a number of morphological alterations as well as a significant reduction in craniofacial size. However, shape analysis revealed that in their study, mice had shorter mandibles and craniums in the anteroposterior dimension. Consumption of a soft diet for 15 generations in a row did not modify the size of the craniofacial structure. Further evidence that changes in shape and size due to different functional loads appeared to be independent was provided by the fact that changes in shape persisted after diets were switched for one generation while size dropped and then returned to baseline. The authors did not mention any changes in the maxilla as a separate anatomic area but they emphasized on cranium [51]. In contrast, Tsolakis et al. [43] used wistar rats as their experimental animals in 2023, and their findings showed that the soft diet groups differed statistically significantly from the hard diet groups in all length measurements. The comparison of all length measurements between the first generation and the third generation of the soft diet group revealed statistically significant changes, despite the fact that there were no differences between the first and second generations of the soft diet groups or between the second and third generations of the soft diet groups. Additionally, in all posterior height linear measurements, there were statistically significant differences between the hard diet groups and all soft diet groups. Furthermore, there were no statistically significant variations in posterior height measurements between the second and third generations of the soft diet groups or between the first and second generations of the soft diet groups. Nevertheless, there were statistically significant differences between the first generation and the third generation of the soft diet groups when all posterior height data were compared. Since there were changes between all generations but the differences were statistically significant two generations later, it was revealed that the long-term mastication of a soft food was capable of affecting the morphology of the mandible. The current study shown that, when comparing soft diet groups to hard diet groups, respectively, mandibular morphology changed on the horizontal and vertical planes, but there was no difference on the transverse plane. It was found out that there were substantial variations between the soft diet groups, mostly in the horizontal linear mandibular measures that comprised condylar points from the first to the second generation. There were significant variations between the second and third generations for all horizontal mandibular measurements. Finally, there were variations between the first and third generations of soft diet groups on both a horizontal and vertical axis for the mandible.

Premaxilla measurements did not alter significantly, according to our study. Between all conceivable comparisons, the anterior portion of the maxilla did not exhibit any appreciable variations. All three generations of the soft diet group showed a narrowing of the dental arch. Only the first generation displayed noticeable alterations on the third molar, while the next two generations displayed noticeable changes on all three molars. This may be explained as a result of the different timing in molars’ eruption. It is known that the first rat molar typically erupts around day 17, the second rat molar on day 20, and the third rat molar on day 33. Since our primary subjects were 30 days of age, it seems logical that only the third molar could be affected by the diet of the first generation. Lastly, our study showed no significant differences on the horizontal plane for maxillary measurements.

Mastication, speech, and respiration are all impacted by craniofacial morphology, specifically jaw morphology. The current study suggests that nutrition can significantly affect craniofacial shape. The soft diet mastication may cause the mandible to become more retrognathic and the maxilla narrower. It appears that genetic information is passed down through the generations and will likely appear to have a substantial impact on mandibular growth. This allowed the mandible to develop more vertically. These findings might imply that the reduced mandible was part of the craniofacial alterations that occurred during human evolution. The current body of literature makes the argument that a smaller mandible may result in crowded teeth, insufficient room for the third molars, and restricted airways. It is crucial to note that recent research has linked the activity of the less-toned muscles in the oropharynx and craniofacial region to a number of chronic conditions, including cardiovascular issues, ADHD, and obstructive sleep apnea [52].

## 5. Conclusions

In conclusion, the results of this study indicate that diet consistency has a significant influence on maxillary and mandibular growth. According to our findings, soft diet habits may result in narrower dental arches, a narrower maxilla, and a retrognathic mandible. The maxilla is mostly affected on the transverse plane by becoming narrower and smaller. On the contrary, the mandible is mostly affected on the horizontal plane by becoming retrognathic and on the vertical plane by becoming shorter in height. This information may be possibly carried over different generations, and it might take more than two generations to be expressed.

## Figures and Tables

**Table 1:** Craniofacial landmarks.

Cephalometric Landmarks	Definition
CoL	Most posterosuperior point on the left mandibular condyle.
CoR	Most posterosuperior point on the right mandibular condyle.
GoL	Most posterior point of the left angular process of the mandible
GoR	Most posterior point of the right angular process of the mandible
Go’L	Point on the most inferior contour of the left angular process on the mandible
Go’R	Point on the most inferior contour of the right angular process on the mandible
CorL	Most posterosuperior point of condylar process on the left side
CorR	Most posterosuperior point of condylar process on the right side
MeL	The most inferior and anterior point of the left lower border of the mandible
MeR	The most inferior and anterior point of the right lower border of the mandible
IdL	Most inferior and anterior point on the left alveolar process of the mandible
IdR	Most inferior and anterior point on the right alveolar process of the mandible
I’L	The most anterior edge of the alveolar bone on the convexity of the left lower incisor.
I’R	The most anterior edge of the alveolar bone on the convexity of the right lower incisor.
I	Point on premaxilla between jawbone and lingual surface of upper incisors
Po	Most posterior point on cranial vault
PL	Most left anterior and lateral point of maxilla.
PR	The most right anterior and lateral point of maxilla.
PM1L	Most left anterior and lateral point of premaxilla
PM1R	Most right anterior and lateral point of premaxilla
PM2L	Most left posterior and lateral point of premaxilla
PM2R	Most right posterior and lateral point of premaxilla
M1L	Most palatal middle point of left 1rst molar
M1R	Most palatal middle point of right 1rst molar
M2L	Most palatal middle point of left 2nd molar
M2R	Most palatal middle point of right 2nd molar
M3L	Most palatal middle point of left 3rd molar
M3R	Most palatal middle point of right 3rd molar

**Table 2:** Reliability performed on 20 randomly selected subjects re-measured 2 weeks apart.

Variables		Cochran’s Alpha
Linear measurements	Go’–MeR	0.810
	Go–MeR	0.787
	CorR–MeR	0.798
	CoR\go’R–MeR	0.803
	CoR–MeR	0.915
	CoR–IdR right	0.929
	CoR–I’R	0.943
	CoR–GoR	0.903
	CoR–Go’R	0.842
	Go’L–MeL	0.871
	GoL–MeL	0.883
	CorL–MeL	0.891
	CoL\Go’L–MeL	0.961
	CoL–MeL	0.857
	CoL–IdL	0.982
	CoL–I’L	0.959
	CoL–GoL	0.912
	CoL–Go’L	0.905
	CoL–CoR	0.843
	Go’L–Go’R	0.823
	MeL–MeR	0.898
	IdL–IdR	0.866
	I–Po	0.819
	PR–PL	0.872
	PM1R–PM1L	0.961
	PM2R–PM2L	0.870
	M1R–M1L	0.767
	M2R–M2L	0.781
	M3R–M3L	0.895

**Table 3:** Results for soft diet groups variables measured, means, and standard deviations.

Diet S (Soft)	Generation			
	1 (*n* = 10)	2 (*n* = 10)	3 (*n* = 10)	Overall
	Mean (SD)	Mean (SD)	Mean (SD)	Mean (SD)
Go’–MeR	18.5 (0.7)	18.3 (0.5)	17.4 (0.2)	18.1 (0.7)
Go–MeR	21.1 (0.3)	20.8 (0.6)	18.8 (0.3)	20.2 (1.1)
CorR–MeR	18.3 (0.6)	18.2 (0.4)	17.5 (0.2)	18.0 (0.6)
CoR\go’R–MeR	1.4 (0.3)	1.2 (0.2)	0.9 (0.1)	1.2 (0.3)
CoR–MeR	22.8 (0.3)	21.6 (0.5)	20.2 (0.2)	21.5 (1.1)
CoR–IdR right	27.0 (0.4)	25.3 (0.4)	24.4 (0.2)	25.6 (1.2)
CoR–I’R	26.1 (0.2)	25.0 (0.2)	24.3 (0.2)	25.1 (0.8)
CoR–GoR	5.81 (0.49)	5.44 (0.64)	5.00 (0.63)	5.34 (0.67)
CoR–Go’R	6.87 (0.69)	6.25 (0.72)	5.75 (0.59)	6.26 (0.77)
Go’L–MeL	19.1 (1.3)	19.8 (0.5)	18.7 (0.2)	19.2 (0.9)
GoL–MeL	20.7 (1.1)	21.0 (0.8)	18.9 (0.2)	20.2 (1.2)
CorL–MeL	18.4 (0.6)	18.4 (0.5)	17.4 (0.2)	18.1 (0.7)
CoL\Go’L–MeL	1.6 (0.2)	1.2 (0.2)	1.0 (0.2)	1.3 (0.3)
CoL–MeL	22.8 (0.3)	21.5 (0.6)	19.7 (0.2)	21.3 (1.3)
CoL–IdL	27.1 (0.4)	25.4 (0.4)	24.3 (0.2)	25.6 (1.2)
CoL–I’L	25.9 (0.3)	24.9 (0.2)	24.3 (0.2)	25.0 (0.7)
CoL–GoL	5.71 (0.49)	5.34 (0.64)	4.90 (0.63)	5.24 (0.67)
CoL–Go’L	6.77 (0.69)	6.15 (0.72)	5.65 (0.59)	6.16 (0.77)
CoL–CoR	17.00 (0.40)	16.62 (0.35)	16.78 (0.32)	16.80 (0.38)
Go’L–Go’R	16.15 (1.07)	15.93 (0.69)	16.23 (0.80)	16.10 (0.85)
MeL–MeR	3.33 (0.82)	3.28 (0.54)	3.33 (0.57)	3.31 (0.64)
IdL–IdR	2.05 (0.20)	1.92 (0.25)	2.00 (0.19)	1.99 (0.22)
I–Po	35.2 (0.61)	35.1 (0.71)	35.8 (0.65)	35.36 (0.65)
PR–PL	8.5 (0.23)	8.3 (0.22)	8.5 (0.20)	8.4 (0.21)
PM1R–PM1L	3.3 (0.12)	3.2 (0.13)	3.3 (0.11)	3.26 (0.12)
PM2R–PM2L	6.3 (0.16)	6.1 (0.16)	6.3 (0.19)	6.23 (0.17)
M1R–M1L	3.4 (0.33)	2.8 (0.32)	2.15 (0.29)	2.68 (0.31)
M2R–M2L	3.56 (0.34)	2.73 (0.34)	2.2 (0.32)	2.73 (0.33)
M3R–M3L	3.57 (0.32)	3.2 (0.31)	2.45 (0.30)	3.40 (0.31)

**Table 4:** Results for hard diet groups variables measured, means, and standard deviations.

Diet H (Hard)	Generation			
	1 (*n* = 10)	2 (*n* = 10)	3 (*n* = 10)	Overall
	Mean (SD)	Mean (SD)	Mean (SD)	Mean (SD)
Go’–MeR	20.4 (0.7)	20.4 (0.3)	20.4 (0.4)	20.4 (0.6)
Go–MeR	21.3 (0.6)	21.3 (0.5)	21.3 (0.7)	21.3 (0.6)
CorR–MeR	18.7 (0.3)	18.7 (0.1)	18.7 (0.5)	18.7 (0.3)
CoR\go’R–MeR	1.8 (0.1)	1.8 (0.5)	1.8 (0.08)	1.8 (0.1)
CoR–MeR	23.5 (0.3)	23.5 (0.7)	23.5 (0.07)	23.5 (0.2)
CoR–IdR right	27.8 (0.2)	27.8 (0.1)	27.8 (0.3)	27.8 (0.2)
CoR–I’R	26.6 (0.3)	26.6 (0.6)	26.6 (0.07)	26.6 (0.3)
CoR–GoR	7.55 (0.55)	7.05 (0.51)	6.67 (0.59)	7.07 (0.65)
CoR–Go’R	8.74 (0.61)	8.17 (0.46)	8.78 (0.74)	8.56 (0.71)
Go’L–MeL	20.3 (0.7)	20.3 (0.3)	20.3 (0.4)	20.3 (0.6)
GoL–MeL	20.7 (0.6)	20.7 (0.5)	20.7 (0.7)	20.7 (0.6)
CorL–MeL	18.9 (0.3)	18.9 (0.1)	18.9 (0.5)	18.9 (0.3)
CoL\Go’L–MeL	1.9 (0.1)	1.9 (0.5)	1.9 (0.08)	1.9 (0.1)
CoL–MeL	23.4 (0.3)	23.4 (0.7)	23.4 (0.1)	23.4 (0.3)
CoL–IdL	27.7 (0.1)	27.7 (0.1)	27.7 (0.2)	27.7 (0.1)
CoL–I’L	26.6 (0.2)	26.6 (0.3)	26.6 (0.2)	26.6 (0.2)
CoL–GoL	7.45 (0.55)	7.00 (0.51)	6.57 (0.59)	7.01 (0.65)
CoL–Go’L	8.64 (0.61)	8.07 (0.46)	8.68 (0.74)	8.46 (0.71)
CoL–CoR	16.86 (0.32)	16.78 (0.42)	16.74 (0.35)	16.79 (0.36)
Go’L–Go’R	16.15 (1.02)	16.31 (0.26)	16.30 (0.80)	16.25 (0.74)
MeL–MeR	3.49 (0.62)	3.05 (0.53)	3.33 (0.57)	3.29 (0.59)
IdL–IdR	1.99 (0.25)	1.92 (0.23)	2.01 (0.16)	1.97 (0.21)
I–Po	34.9 (0.72)	35.3 (0.61)	35.7 (0.65)	35.3 (0.66)
PR–PL	8.4 (0.26)	8.1 (0.24)	8.6 (0.22)	8.36 (0.24)
PM1R–PM1L	3.2 (0.15)	3.0 (0.13)	3.3 (0.16)	3.16 (0.14)
PM2R–PM2L	6.2 (0.18)	6.5 (0.12)	6.1 (0.14)	6.16 (0.14)
M1R–M1L	4.0 (0.29)	4.2 (0.31)	4.3 (0.33)	4.16 (0.31)
M2R–M2L	4.2 (0.34)	4.1 (0.34)	4.4 (0.32)	4.3 (0.33)
M3R–M3L	4.6 (0.35)	4.8 (0.33)	4.9 (0.31)	4.76 (0.31)

**Table 5:** Bonferroni corrected (multiple comparisons). *p* values.

	S1–H1	S2–H2	S3–H3	S1–S2	S1–S3	S2–S3	H1–H2	H1–H3	H2–H3
Right-Side Mandible									
Go’R–MeR	**<0.001**	**<0.001**	**<0.001**	>0.999	**0.007**	**0.006**	0.079	0.065	0.137
GoR–MeR	>0.999	0.258	**<0.001**	>0.999	**0.010**	**0.041**	0.080	0.079	0.087
CorR–MeR	0.096	**0.036**	**<0.001**	>0.999	**0.004**	**0.027**	0.091	0.068	0.15
CoR\Go’R–MeR	**0.047**	**<0.001**	**<0.001**	0.062	**0.008**	0.062	0.085	0.094	0.093
CoR–MeR	**<0.001**	**<0.001**	**<0.001**	**0.021**	**0.011**	**0.031**	0.089	0.088	0.089
CoR–IdR	**<0.001**	**<0.001**	**<0.001**	**0.010**	**0.026**	**0.009**	0.109	>0.999	0.236
CoR–I’R	**<0.001**	**<0.001**	**<0.001**	**0.034**	**0.003**	**0.013**	0.201	>0.999	0.311
CoR–GoR	**<0.001**	**<0.001**	**<0.001**	>0.999	**0.007**	0.320	0.204	0.150	0.750
CoR–Go’R	**<0.001**	**<0.001**	**<0.001**	0.422	**0.003**	0.248	0.148	0.300	0.781
Left-Side Mandible									
Go’L–MeL	**0.001**	0.533	**<0.001**	0.091	**0.029**	**0.005**	0.101	0.069	0.143
GoL–MeL	>0.999	>0.999	**<0.001**	>0.999	**0.015**	**0.028**	0.083	0.083	0.093
CorL–MeL	**0.008**	**0.013**	**<0.001**	>0.999	**0.007**	**0.032**	0.097	0.072	0.023
CoL\Go’L–MeL	**0.005**	**<0.001**	**<0.001**	**0.042**	**0.012**	**0.035**	0.086	0.096	>0.084
CoL–MeL	**0.001**	**<0.001**	**<0.001**	**0.036**	**0.009**	**0.028**	0.090	0.092	0.095
CoL–IdL	**<0.001**	**<0.001**	**<0.001**	**0.008**	**0.031**	**0.016**	0.123	>0.999	0.242
CoL–I’L	**<0.001**	**<0.001**	**<0.001**	**0.027**	**0.005**	**0.007**	0.231	>0.999	0.371
CoL–GoL	**<0.001**	**<0.001**	**<0.001**	>0.999	**0.01**	0.240	0.234	0.210	0.624
CoL–Go’L	**<0.001**	**<0.001**	**<0.001**	0.232	**0.004**	0.568	0.418	0.420	0.511
Transverse Mandible									
CoL–CoR	>0.999	0.974	>0.999	0.088	0.710	>0.999	>0.999	>0.999	>0.999
Go’L–Go’R	>0.999	>0.999	>0.999	>0.999	>0.999	>0.999	>0.999	>0.999	>0.999
MeL–MeR	>0.999	>0.999	>0.999	>0.999	>0.999	>0.999	0.470	>0.999	>0.999
IdL–IdR	>0.999	>0.999	>0.999	0.7345	>0.999	>0.999	>0.999	>0.999	>0.999
Maxilla									
I–Po	>0.999	>0.999	>0.999	>0.999	>0.999	>0.999	>0.999	>0.999	>0.999
Transverse Maxilla									
PR–PL	>0.999	>0.999	>0.999	0.618	>0.999	>0.999	>0.999	>0.999	>0.999
PM1R–PM1L	>0.999	>0.999	>0.999	>0.999	>0.999	>0.999	>0.999	>0.999	>0.999
PM2R–PM2L	>0.999	>0.999	>0.999	>0.999	>0.999	>0.999	>0.999	>0.999	>0.999
M1R–M1L	>0.999	>0.999	>0.999	0.7345	>0.999	>0.999	>0.999	>0.999	>0.999
M2R–M2L	>0.999	>0.999	>0.999	>0.999	>0.999	>0.999	>0.999	>0.999	>0.999
M3R–M3L	>0.999	>0.999	>0.999	>0.999	>0.999	>0.999	>0.999	>0.999	>0.999
PR–PL	>0.999	>0.999	>0.999	0.894	>0.999	>0.999	>0.999	>0.999	>0.999
PM1R–PM1L	>0.999	0.891	>0.999	>0.999	>0.999	>0.999	>0.999	>0.999	>0.999
PM2R–PM2L	>0.999	>0.999	>0.999	>0.999	>0.999	>0.999	>0.999	>0.999	>0.999
M1R–M1L	0.057	**<0.001**	**<0.001**	0.077	**0.008**	0.072	0.095	0.078	0.084
M2R–M2L	0.068	**<0.001**	**<0.001**	0.059	**0.012**	0.034	0.079	0.094	0.093
M3R–M3L	**0.040**	**<0.001**	**<0.001**	0.062	**0.006**	0.055	0.083	0.085	0.088

## Data Availability

The corresponding author will provide the datasets used and/or analyzed during the current work upon reasonable request.
